# Tracking Changes in Maximal Oxygen Consumption with the Heart Rate Index in Female Collegiate Soccer Players

**DOI:** 10.2478/hukin-2014-0065

**Published:** 2014-10-10

**Authors:** Michael R. Esco, Ronald L. Snarr, Andrew Flatt, Matthew Leatherwood, Adam Whittaker

**Affiliations:** 1 Department of Kinesiology, University of Alabama, Tuscaloosa, AL.; 2 Human Performance Laboratory, Department of Physical Education and Exercise Science, Auburn University at Montgomery, Montgomery, AL.

**Keywords:** athletes, women, sports, aerobic fitness

## Abstract

The purpose of this study was to determine if the HRindex Method (VO_2max_ = [6 x HRindex – 5] x 3.5, where HRindex = HRmax/HRrest) was accurate for tracking changes in VO_2max_ following 8-weeks of endurance training among collegiate female soccer players. Predicted VO_2max_ via the HRindex Method and observed VO_2max_ from a maximal exercise test on a treadmill were determined for a group of female soccer athletes (n = 15) before and following an 8-week endurance training protocol. The predicted (pVO_2max_) and observed (aVO_2max_) values were compared at baseline and within 1-week post-training. Change values (i.e., the difference between pre to post) for each variable were also determined and compared. There was a significant difference between aVO_2max_ before (43.2 ± 2.8 ml·kg·min^−1^) and following (46.2 ± 2.1 ml·kg·min^−1^) the 8-week training program (p < 0.05). However, pVO_2max_ did not significantly change following training (pre = 43.4 ± 4.6 ml·kg·min^−1^, post = 42.9 ± 4.1 ml·kg·min^−1^, p = 0.53). Furthermore, the correlation between the change in aVO_2max_ and the change in pVO_2max_ was trivial and non-significant (r = 0.30, p = 0.28). The HRindex Method does not appear to be suitable for predicting changes in VO_2max_ following 8-weeks of endurance training in female collegiate soccer players.

## Introduction

Maximal oxygen consumption (VO_2max_) is an important physiological determinate of athletic performance among many team sports. For example, oxidative phosphorylation accounts for the majority of energy production during a soccer game (Bangsbo, 1994). In fact, VO_2max_ has been shown to be an important contributor to repeated sprint ability, total distance covered, and the number of ball contacts made during soccer-play (Dawson et al., 1993; Helgerud et al., 2001; McMahon and Wenger, 1998). Recently, Jones et al. (2013) demonstrated that maximal aerobic capacity was an important factor in aiding recovery between intermittent sprinting in professional soccer players. Wisloff et al. (1998) demonstrated a significant difference in VO_2max_ between the top and lower placed teams of elite competition. Thus, success in team sports like soccer may depend heavily on well developed aerobic energy systems among players.

Gold standard measures of VO_2max_ primarily occur in exercise physiology laboratories and involve specialized equipment operated by trained personnel. Precise measures are often not readily available to sport practitioners. Instead, field tests to estimate VO_2max_ exist in practical settings with minimal equipment. Many applied tests use the heart rate (HR) as a simple physiological parameter to predict VO_2max_ (Esco et al., 2011; Haller et al., 2013; Macsween, 2002; Marsh, 2012; Wicks et al., 2011). Most of the established models employ the use of submaximal HR as a prediction variable. The disadvantage of this method comes with the assumption of a uniform maximal heart rate (HR_max_) across age and an absolute linear response in the HR and VO_2_ from rest to maximal exertion (Haller et al., 2013). However, the relationship between age and the HRmax is inconsistent (Robergs and Landwehr, 2002), and nonlinear responses in the HR and VO_2_ during progressive exercise have been documented (Bodner and Rhodes, 2000; Zoladz et al., 2007). Thus, submaximal HR-based prediction models often carry a wide range of estimation error when compared to laboratory-derived VO_2max_ (Macsween, 2002; Marsh, 2012).

Recently, Wicks et al. (2011) developed the HRindex Method that predicted submaximal and maximal oxygen consumption from the ratio of the exercise heart rate (HR_absolute_) to resting heart rate (HR_rest_). It was determined that the HRindex Method was capable of accurately predicting VO_2max_ when the maximal heart rate (HR_max_) was utilized as the HR_absolute_, independent of testing mode, age, sex, fitness, and body weight (Wicks et al., 2011). It is due to its simplicity that this method may be attractive for estimating VO_2max_ within athletic field settings. However, there is limited cross-validation research in this area. Two studies have shown that the HRindex Method resulted in a wide-range of individual error in untrained men (Esco et al., 2011; Haller et al., 2013). However, there are no available studies to determine the accuracy of the HRindex Method for tracking changes in VO_2max_ following a period of endurance training, particularly among female athletes. This research is needed as differences among the prediction variables of the HRindex Method (i.e., HR_rest_ and HR_max_) and VO2max have been reported between men versus women and between athletes versus non-athletes (Dela et al., 1992; Faulkner et al., 1997; Pakkala et al., 2005; Vassalle et al., 2013). Furthermore, the HR_rest_ and HR_max_ may or may not change in response to an increased VO_2max_ (An et al., 2006; Cornelissen et al., 2010; Ekblom, 1968; Oliveira et al., 2013; Raczak et al., 2006; Uusitalo et al.,1998). Therefore, the purpose of this study was to determine if the HRindex Method was accurate for tracking changes in VO_2max_ following 8-weeks of endurance training among collegiate female soccer players. Based on the previous findings in non-athletic men (Esco et al., 2011; Haller et al., 2011), it was hypothesized that the HRindex Method would provide an accurate assessment of VO_2max_ at baseline and following training among the entire group (i.e., no significant mean differences between predicted and observed values), but that it would result in a wide range of individual error at both time points.

## Material and Methods

### Subjects

Fifteen female soccer players (age = 21.5 ± 1.8 years; body height = 167.2 ± 6.0 cm; body mass = 64.2 ± 7.4 kg) from the National Association of Intercollegiate Athletes (NAIA) participated in this study and provided written informed consent. The study was approved by the Institutional Review Board at the Auburn University at Montgomery for research involving human subjects. All subjects were free from cardiovascular, pulmonary, and metabolic diseases. Pre- and post-training data collection was conducted within an exercise physiology laboratory in the morning hours between 7 am and 11 am on any weekday as close as possible to awakening from sleep. Before each day of testing, the athletes were required to refrain from the consumption of food or caffeine for at least 8 hours prior and to avoid strenuous exercise and alcohol consumption for 24 hours prior.

### Maximal Graded Exercise Test

Each subject performed a maximal graded exercise test using a Trackmaster treadmill (Full Vision, Inc., Carrollton, TX) and a calibrated ParvoMedics TrueOne^®^ 2400 metabolic cart (ParvoMedics Inc., Sandy, UT). The Bruce protocol was employed, which began at 1.7 mph at 10% grade with increasing speed and grade (i.e., 2.5 mph at 12%, 3.4 mph at 14%, 4.2 mph at 16%, 5.0 mph at 18%, etc.) every 3 minutes until test termination. Observed VO_2max_ (aVO_2max_) was achieved if two of the following criteria occurred: a plateau in VO_2_ (< 2.0 mL·kg^−1^·min^−1^) with an increasing work rate; the respiratory exchange ratio equal to or greater than 1.15; the HR within 10 beats of age predicted maximum (220 – age); or volitional fatigue.

### Heart Rate Measures

Heart rate data was collected with a Polar F11 HR Monitor (Polar Electro Oy, Kemple, Finland). Before the GXT, the subjects assumed a supine position for 5-minutes in a quiet, climate controlled, dimly lit exercise physiology laboratory. The lowest heart rate during the last 1-minute of the supine period was recorded as the HR_rest_. The Polar HR Monitor remained on the subject during the GXT and the HR value that corresponded to VO_2max_ was recorded as the HR_max_.

### Heart Rate Index Method

Comprehensive details concerning the development of the HRindex equation may be found in the study of Wicks et al. (2011). Briefly, the HRindex is determined as the ratio between the HR at a selected level of exercise intensity (i.e., HR_absolute_) and HRrest. When determining VO_2max_, the HR_max_ is utilized as the HR_absolute_ (Wicks et al., 2011). Thus, this paper utilized the following equation for predicted VO_2max_ (pVO_2max_):

$$\begin{array}{l} {\text{pVO}}_{2\text{max}}=[{\text{HR}}_{\text{index}}\cdot 6)-5]\cdot 3.5\;\text{ml}\cdot {\text{kg}}^{-1}\cdot {\text{min}}^{-1}\\ {\text{HR}}_{\text{index}}={\text{HR}}_{\text{absolute}}/{\text{HR}}_{\text{rest}} \end{array}$$

Where HR_absolute_ = HR_max_ recorded during the GXT; and HR_rest_ = the lowest HR value recorded during the resting condition (as described above).

### Training program and post-data collection

Following the testing procedures, the athletes followed an 8-week endurance training program that was designed by the team’s coach and consisted of an unstructured mixture of high-intensity interval and continuous aerobic exercise for approximately 1 hour per session. Exercise training was performed at least 4 days per week. According to the coach, the primary objective of the program was to improve the team’s average VO_2max_. The researchers of the study had little involvement with the development or implementation of the team’s exercise program. The investigators tested the athletes in the laboratory for aVO_2max_ and pVO_2max_ at baseline (pre) and within 1-week following (post) the 8-week endurance training program.

### Statistical Analysis

Statistical analyses were performed using PASW/SPSS version 18.0 (Cary, NC). Means and standard deviations (SD) were determined for the observed and predicted VO_2max_ values at pre- and post-training. A 2 (observed versus predicted) by 2 (pre versus post) mixed design analysis of variance (ANOVA) procedure was used to determine if there were differences between the VO_2max_ values at preand post-training. If the ANOVA revealed significance, the Fisher’s least significant difference (LSD) post-hoc test was used to further examine the differences in VO_2max_ values. The Cohen’s d statistic was calculated to determine the effect size of the mean differences. In addition, bias between criterion and predicted (Bias = pVO_2max_ – aVO_2max_) values was determined at pre (Bias-PRE) and post (Bias-POST) training. The changes in observed and predicted VO_2max_ from pre to post were determined as follows: ΔaVO_2max_ = aVO_2max_POST – aVO_2max_PRE; and ΔpVO_2max_ = pVO_2max_POST – pVO_2max_PRE, respectively. Zero-order correlation procedures determined the relationship between the observed and predicted VO_2max_ values at PRE and POST, and between ΔaVO_2max_ and ΔpVO_2max_. The standard error of estimate (SEE) of the predicted values was also determined at pre and post. Furthermore, the method of Bland-Altman was carried out to determine the limits of agreement between the observed and prediction methods at both time points.

## Results

At baseline, HR_rest_, HR_max_ and HRindex (i.e., HR_max_ / HR_rest_) were 62.9 ± 2.8 beats·min^−1^, 182.0 ± 9.1 beats·min^−1^, and 2.9 ± 0.2, respectively. Following the 8-weeks of endurance training, HR_rest_, HR_max_ and HRindex were 63.1 ± 3.2 beats·min^−1^, 181.1 ± 8.4 beats·min^−1^, and 2.9 ± 0.2, respectively. There were no significant differences in the pre and post values for HR_rest_ (p = 0.76), HR_max_ (p = 0.50), and HRindex (p = 0.52).

Table 1 displays the mean values for pre and post-training aVO_2max_ and pVO_2max_, Bias-PRE and Bias-POST, as well as ΔaVO_2max_ and ΔpVO_2max_. The 8-week training program increased observed VO_2max_, as aVO_2max_POST was 3 ml·kg^−1.^min^−1^ higher compared to aVO_2max_PRE (p < 0.05, Cohen’s d = 1.21, Table 1). However, there was no significant difference between pVO_2max_PRE and pVO_2max_POST (p = 0.53, Cohen’s d = 0.11, Table 1). The predicted and observed values were not significantly different at baseline (p = 0.81 Cohen’s d = 0.05, Table 1), but were significantly different at follow-up testing (p < 0.05, Cohen’s d = 0.78, Table 1).

Zero-order correlation procedures found a moderate non-significant relationship between aVO_2max_PRE and pVO_2max_PRE (r = 0.48, p = 0.08), a trivial non-significant relationship between aVO_2max_POST and pVO_2max_POST (r = 0.30, p = 0.27), and a trivial non-significant relationship between ΔaVO_2max_ and ΔpVO_2max_ (r = 0.30, p = 0.28). The SEE for pVO_2max_PRE was 3.81 ml·kg^−1.^min^−1^ and for pVO_2max_POST was 4.86 ml·kg^−1.^min^−1^, which corresponded to 8.9% of aVO_2max_PRE and 10.5% of aVO_2max_POST, respectively.

**Figure 1:**
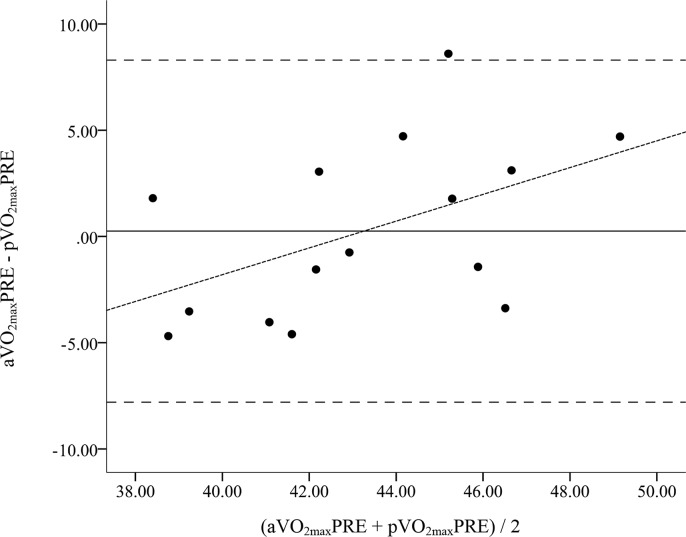
Bland-Altman Plots comparing the VO_2max_ estimation by the HRindex method (pVO_2max_PRE) with observed VO_2max_ (aVO_2max_PRE) at baseline The solid lines represent the mean difference. The large dashed outside lines represent the upper and lower limits of agreement (95% confidence interval of the mean difference). The small dashed regression line represents the trend between the differences of methods and the mean of both methods

**Figure 2:**
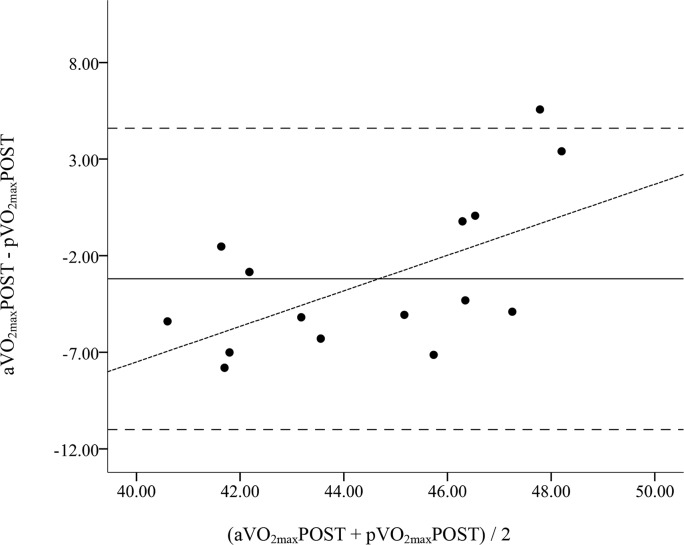
Bland-Altman Plots comparing the VO_2max_ estimation by the HRindex method (pVO_2max_POST) with observed VO_2max_ (aVO_2max_POST) following 8-weeks of endurance training The solid lines represent the mean difference. The large dashed outside lines represent the upper and lower limits of agreement (95% confidence interval of the mean difference). The small dashed regression line represents the trend between the differences of methods and the mean of both methods.

Bland-Altman Plots comparing the pre and post values are shown in Figures 1 and 2, respectively. The 95% confidence intervals (CI) for pVO_2max_PRE ranged from 7.7 ml·kg^−1.^min^−1^ below to 8.3 ml·kg^−1^·min^−1^ above the mean difference of 0.3 ml·kg^−1^·min^−1^, with a significant trend (r = 0.50, p < 0.05, Figure 1). The 95% CI for pVO_2max_POST ranged from 11.0 ml·kg^−1^·min^−1^ below to 4.6 ml·kg^−1^·min^−1^ above the mean difference of −3.2 ml·kg^−1^·min^−1^, with a significant trend (r = 0.60, p < 0.05, Figure 2).

## Discussion

Accurate methods for predicting VO_2max_ in field settings, especially in response to endurance training are needed by practitioners and coaches of sports teams. This study sought to determine if the HRindex Method was a suitable means for tracking changes in VO_2max_ in a group of female collegiate soccer players following an 8-week endurance training program that had been designed by the team’s coach. The 8-week training program significantly improved aVO_2max_ by 7% from baseline, as the difference between the pre and post observed values was significant and the Cohen’s d statistic indicated a large effect size (Cohen, 1988). However, no significant change in pVO_2max_ following training was found. When comparing the baseline values, there were no significant differences and a moderate, non-significant correlation between the observed and predicted VO_2max_ values. However, there were significant differences and a trivial, non-significant correlation between the observed and predicted VO_2max_ values. Furthermore, the SEE increased from 8.9% to 10.5% of observed VO_2max_ from baseline to post-training. In addition, Bland-Altman plots revealed wide limits of agreement at pre and post time points, indicating wide individual error. The significant trends between the difference of the 2 methods (y-axes) and the mean of the 2 methods (x-axes) of the Bland-Altman Plots at both time points suggested a greater overestimation of VO_2max_ within individuals who had observed values lower than the group mean. Therefore, the HRindex was not suitable for tracking changes in VO_2max_ in female soccer players following 8-weeks of endurance training and resulted in a wide range of individual prediction error at both time points.

The HRindex equation was developed by Wicks et al. (2011) as a simple method for predicting oxygen uptake with the ratio of exercise HR to resting HR. The equation was developed from 220 group mean data sets extracted from 60 published exercise studies and apparently explained 99.1% of the variation in oxygen uptake in the study (Wicks et al., 2011). Unfortunately, cross-validation analyses were not performed (Wicks et al., 2011). Future study was warranted to establish prediction errors for individuals and specific groups.

Two previous investigations are available that determined the accuracy of the HRindex Method among groups of non-athletic men (Esco et al., 2011; Haller et al., 2013). Esco et al. (2011) showed large limits of agreement when comparing VO_2max_ determined in the laboratory and predicted via the HRindex equation in a large sample of college-age men. Haller et al. (2013) demonstrated that the HRindex Method significantly underestimated VO_2max_ and also produced large individual prediction errors across various exercise testing protocols in a group of aerobically fit, young men (Haller et al., 2013). The current investigation was the first to establish the accuracy of the HRindex method in female athletes and to determine its suitability for tracking changes in VO_2max_ following training.

According to the Fick equation, oxygen consumption is the product of cardiac output (Q) and an arteriovenous oxygen difference (a-vO_2_diff). An increase in VO_2max_ following training has been shown to be a result of an increase in both of these components (Powers and Howley, 2012). However, the primary contribution of an increase in VO_2max_ between the central (i.e., Q) and peripheral (i.e., avO_2_diff) components depends on training duration (Ekblom, 1968). Classic research has shown that the improvement in VO_2max_ within the first few months of endurance training is primarily due to an increase in stroke volume mediated by an increase in systemic blood flow (Ekblom, 1968). Further improvements in VO_2max_ with longer periods of training are due to peripheral changes of enhanced oxygen extraction with an increased capillary density of skeletal muscle (Ekblom, 1968). In the presence of improved stroke volume, the increased filling time requirement between each heart beat (i.e., a longer diastolic phase) results in a lower HR_rest_ (Powers and Howley, 2012). Since a change in HR_max_ following training is not typical, it could be theorized that the difference between resting and maximal HR would increase after a period of chronic endurance training. If this occurred, then a larger HRindex (i.e., HR_max_ – HR_rest_) and a greater pVO_2max_ derived by the equation would have resulted. However, a change in the HRindex following training was not demonstrated in the current study mainly because neither the prediction variables (i.e., HR_rest_ and HR_max_) changed from pre to post. Therefore, pVO_2max_ did not increase following the training program despite an increase in aVO_2max_. As noted previously, the HR is a parameter of Q that increases or decreases in response to a respective decrease or increase in stroke volume. As a result of no change in the HR parameters, we can conclude no subsequent change in stroke volume took place in the studied sample. Therefore, perhaps the improvement in aVO_2max_ following the training program was primarily due to an improvement in peripheral oxygen extraction (i.e., increased a-vO_2_diff), which was not accounted for in the HRindex equation. Though this is a reasonable explanation of the findings, it is only speculative as blood gases were not analyzed in this investigation. At any rate, the HRindex equation did not reflect improvements in observed VO_2max_ in the group of competitive female collegiate athletes.

Another explanation of the findings may be due to how the HR_rest_ was determined in the current study. Among the 60 studies reviewed by Wicks et al. (2011) that were used to develop the HRindex equation, only 12 documented how the HR_rest_ was recorded. Therefore, comparing how the HR_rest_ was determined in the current study to all of the studies reviewed by Wicks et al. (2011) is impossible. Currently, there are no accepted standard recommendations for recording the HR_rest_, despite its importance as a prognostic variable related to cardiovascular disease risks (Fox et al., 2007). Standardization of methods could possibly decrease prediction error associated with the HRindex equation and enhance the utility of the HR_rest_ for predicting VO_2max_. Future research in this area is needed.

Although aerobic power is an important contributor to soccer performance, it should not be the exclusive focus when testing athletes from this population. The physiological demands of the sport require athletes to be proficient in several aspects of physical fitness, such as anaerobic power, agility, speed, etc. (Bangsbo et al., 2006). Therefore, other field tests may also be important for tracking changes in fitness variables that are related to soccer. The Yo-Yo Intermittent Recovery Test has been shown to relate more strongly to specific aspects of soccer performance (e.g., high intensity running during a game) compared to VO_2max_ (Bangsbo et al., 2008; Krustrup et al., 2003). However, assessing a player’s VO_2max_ certainly has value, but due to the results of the current study practitioners should consider other tests when predicting aerobic power in field settings. For example, the 20-m shuttle run test and the 20-m square shuttle run test revealed SEE values of 2.97 ml·kg^−1^·min^−1^ (6.7% of observed VO_2max_) and 2.39 ml·kg^−1^·min^−1^ (5.4% of observed VO_2max_), respectively, in another group of female collegiate soccer players, which were quite lower compared to the SEE values of the current study (Green et al., 2013).

It should be noted that the investigators of the study did not have control over the training program. This could be considered a limitation since the training load could not be quantified hence examining the effects of the exercise program on changes in VO_2max_ was difficult. However, the primary objective of the study was to determine the accuracy of the HRindex Method for predicting changes in VO_2max_. Therefore, not quantifying the training load of the exercise program did not influence the study’s findings.

In conclusion, this study sought to determine if the HRindex Method was suitable for tracking changes in VO_2max_ in a group of female collegiate soccer players following an 8-week endurance training program. To perform this method, all that is required is an exercise ergometer, a method of measuring the HR, and a subject willing to perform a maximal exercise test. It is because of this simplicity that the HRindex Method could be attractive for estimating VO_2max_ in field settings among athletes. However, the results of this study indicated that the HRindex was not valid in tracking changes in VO_2max_ following training, and resulted in wide individual prediction error at the pre and post-training measurement periods in a group of collegiate female soccer athletes. Therefore, sports practitioners who work with this population should consider other established field methods for tracking changes in VO_2max_ following a period of endurance training.

## Figures and Tables

**Table 1:** Baseline, Post, and Change VO_2max_ values (ml·kg^−1^·min^−1^) for observed and predicted values (n = 15)

		Mean	SD
Baseline	aVO_2max_PRE	43.2	2.8
	pVO_2max_PRE	43.4	4.6
	Bias-PRE	0.3	4.1
Post	aVO_2max_POST	46.2^*^	2.1
	pVO_2max_POST	42.9^†^	4.1
	Bias-POST	−3.2	4.0
Change	ΔaVO_2max_	3.0	1.8
	ΔpVO_2max_	−0.5^†^	3.0
	Bias-Δ	−3.4	3.0

aVO_2max_PRE = observed VO_2max_ at baseline, pVO_2max_PRE = predicted VO_2max_ at baseline, Bias-PRE = the difference between pVO_2max_PRE and aVO_2max_PRE, aVO_2max_POST = observed VO_2max_ at post, pVO_2max_ = predicted VO_2max_ at post, Bias-POST = the difference between pVO_2max_POST and aVO_2max_POST, ΔaVO_2max_ = change in observed VO_2max_ from baseline to post, ΔpVO_2max_ = change in predicted VO_2max_ from baseline to post, Bias-Δ = the difference between ΔpVO_2max_ and ΔaVO_2max_.

* Significantly different from PRE values (p < 0.05).

† Significantly different compared to observed values
